# Effect of *In Vitro* Syncytium Formation on the Severity of Human Metapneumovirus Disease in a Murine Model

**DOI:** 10.1371/journal.pone.0120283

**Published:** 2015-03-24

**Authors:** Laetitia Aerts, Marie-Hélène Cavanagh, Julia Dubois, Julie Carbonneau, Chantal Rhéaume, Sophie Lavigne, Christian Couture, Marie-Ève Hamelin, Guy Boivin

**Affiliations:** 1 Centre de Recherche en Infectiologie of the Centre Hospitalier Universitaire de Québec and Université Laval, Quebec, Canada; 2 Anatomopathologie et cytologie, Institut Universitaire de Cardiologie et de Pneumologie de Québec and Université Laval, Quebec City, QC, Canada; University of Iowa, UNITED STATES

## Abstract

Human metapneumovirus (HMPV) is an important cause of acute respiratory tract infections (ARTI) in children, elderly individuals and immunocompromised patients. *In vitro*, different HMPV strains can induce variable cytopathic effects ranging from large multinucleated syncytia to focal cell rounding. In this study, we investigated the impact of different *in vitro* phenotypes of two HMPV strains on viral replication and disease severity in a BALB/c mouse model. We first generated two recombinant GFP-expressing HMPV viruses: C-85473, a syncytium-inducing strain (rC-85473) belonging to the A1 subtype and CAN98-75, a focal cell rounding-inducing strain (rCAN98-75) of the B2 subtype. We subsequently exchanged the F genes of both strains to create the chimeric viruses rC-85473_F and rCAN98-75_F. We demonstrated that the F protein was the sole protein responsible for the syncytium phenotype and that viruses carrying a syncytium-inducing F protein replicated to significantly higher titers *in vitro*. *In vivo*, however, the virulence and replicative capacity of the different HMPV strains did not appear to be solely dependent on the F gene but also on the viral background, with the strains containing the C-85473 background inducing more weight loss as well as increased lung viral titers, pro-inflammatory cytokines and inflammation than strains containing the CAN98-75 background. In conclusion, the F protein is the main determinant of syncytium formation and replication kinetics *in vitro*, although it is not the only factor implicated in HMPV disease severity in mice.

## Introduction

Human metapneumovirus (HMPV) is a major cause of acute respiratory tract infections (ARTI) in children, elderly individuals and immunocompromised patients [1]. For instance, HMPV accounts for 10 to 15% of all hospitalizations for ARTI in children [2]. Clinical signs associated with HMPV are similar to those associated with human respiratory syncytial virus (HRSV), ranging from mild respiratory problems to bronchiolitis and pneumonia [3]. Based on phylogenetic analysis of the F and G genes, HMPV strains can be classified into two main lineages (A and B), each containing 2 or 3 sub-lineages (A1, A2a, A2b, B1 and B2) [4,5]. Whether these HMPV lineages are associated with different clinical outcomes remains unclear; some groups found no evidence for differential severity between HMPV lineages [6,7], whereas others suggested more severe clinical disease associated with HMPV-A [8] or HMPV-B [9,10] strains. However, a recent study by our group, suggested that viral load rather than HMPV lineage was an independent risk factor for severe disease [11].

**Fig 1:**
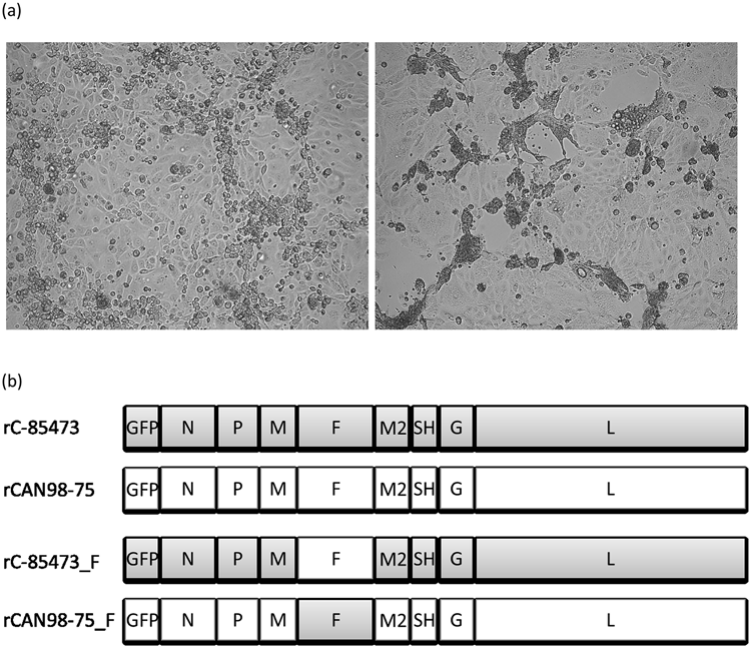
Cytopathic effects of HMPV strains and recombinant HMPV viruses. (a) microscopic images of cytopathic effects induced by HMPV infection of LLC-MK2 monolayers. CAN98–75 (B2) induces focal cell-rounding (left) whereas C-85473 (A1) induces multinucleated syncytia (right). Magnification = 10x. (b) Representation of the genomes of the 4 recombinant viruses used in this study; rC-85473 and rCAN98–75 represent the wild-type strains, rC-85473_F represents the chimeric rC-85473 strain in which the F gene has been replaced with that of CAN98–75 and rCAN98–75_F represent the chimeric rCAN98–75 in which the F gene has been replaced with that of C-85473.

HMPV culture is notoriously difficult. Viruses have to be cultured several weeks before cytopathic effects (CPE) occur and regular addition of exogenous trypsin is required. In addition, CPE differ greatly between strains ranging from typical HRSV-like syncytia to focal cell rounding in tertiary monkey kidney cells such as LLC-MK2 cells (Fig. 1a).

HMPV is a member of the *Pneumovirinae* subfamily within the *Paramyxoviridae* family [12]. Paramyxovirus entry into the host cells occurs through fusion of the cell membrane with the viral envelope. This fusion is mediated by viral surface glycoproteins. Membrane fusion of members of the *Pneumovirinae* subfamily (including HRSV and HMPV) is unique among paramyxoviruses, because the fusion (F) glycoprotein alone is sufficient for membrane fusion to occur without the requirement of an additional attachment glycoprotein [13,14]. The paramyxovirus F protein is synthesized as an inactive F_0_ precursor protein that requires proteolytic cleavage into 2 disulfide-linked subunits (F_1_ and F_2_) to be activated and capable of inducing membrane fusion. Proteolytic cleavage reveals a hydrophobic fusion peptide located at the N-terminus of the F_1_ subunit, which is inserted into target cell membranes to initiate folding of two heptad repeats within the F_1_ subunit, HRA and HRB, into an irreversible six-helix bundle. These conformational changes result in the formation of a fusion pore [15].

Although it is generally recognized that activated F protein-mediated cell-cell fusion is the cause of syncytium formation, the exact reasons why some strains induce large syncytia and others do not remain to be established. In addition, some HMPV strains are dependent on low-pH for membrane fusion *in vitro* [16]. Several studies have tried to elucidate which amino acids could be responsible for this pH-dependency. One group proposed that pH-dependency is dependent on the HMPV lineage [17]. Using 2 subtype A strains and 2 subtype B strains, they suggested that Gly_294_ was responsible for pH-dependency of some subtype A viruses and that subtype A viruses carrying Glu_294_ did not induce syncytium at any pH. On the other hand, both subtype B viruses with Glu at position 294 proved to induce syncytium in a pH-independent fashion. They proposed that a tetrad of variable amino acids at positions 294, 296, 396 and 404 was likely involved in protonation of conserved histidine residues at positions 368 and 435 in pH-dependent strains [18]. Chang et al, on the other hand, used the prototype pH-dependent strain CAN97–83 (A2) to propose that the tetrad at positions 294, 296, 396 and 438 was involved in destabilizing the histidine at position 435 [19]. However, due to the very limited number of strains analyzed in these studies, it is very difficult to generalize these results. More importantly, the relevance of pH-dependency or—independency on severity of HMPV infection remains unexamined.

Moreover, within our collection of HMPV isolates, we have encountered type A strains that do induce syncytium at neutral pH and type B strains that do not. By comparing the type A1 strain C-85473 (syncytium phenotype) and the type B2 strain CAN98–75 (focal cell rounding phenotype) (Fig. 1a), we initially observed increased replication kinetics *in vitro* and increased virulence in BALB/c mice with the former strain. We then generated the recombinant viruses from these strains containing GFP as a reporter gene and we further swapped the F genes in both viruses (Fig. 1b). Herein, we report that replacing the F gene of a focal cell rounding-inducing strain with that of a syncytium-forming strain, or vice versa, is sufficient to alter the phenotype of the strain *in vitro*. We also investigated replication kinetics of all 4 recombinant strains in cell culture and in the lungs of infected BALB/c mice. We found that HMPV strains carrying the syncytium-inducing F protein replicated to higher titers *in vitro* than non-syncytium F protein, but that the F protein was not the only contributing factor to HMPV disease severity in animals.

## Materials and Methods

### Cells and HMPV strains

LLC-MK2 cells (ATCC CCL-7) were maintained in minimal essential medium (MEM) (Life Technologies) supplemented with 10% fetal bovine serum (FBS) (Wisent).

BSR-T7/5 cells (a gift from Dr Ursula Buchholz at the NIAID in Bethesda, MD) were cultured in MEM supplemented with 10% FBS (Wisent), 1% Non-essential amino acids (NEAA) (Life Technologies), 10 mM HEPES (sigma), 1% penicillin / streptomycin (Wisent) and 0.2 mg/ml geneticin (G418, Life Technologies). The HMPV group A strain C-85473 and group B strain CAN98–75 were grown on LLC-MK2 cells in OptiMEM (Life technologies) supplemented with 0.0002% trypsin (Sigma). Virus stocks were concentrated on Amicon columns (Fisher Scientific) as previously described [20].

### Virus quantification

Viral titers were determined by 10-fold serial dilutions of recombinant virus or lung homogenates in 24-well plates containing LLC-MK2 cells as previously reported [21]. Virus titers were reported as 50% tissue culture infectious doses (TCID_50_) per ml. TCID_50_ values were calculated by the Reed and Muench method.

Alternatively, the number of PFU/ml was calculated to determine the MOI for *in vitro* infection experiments (syncytium assay, real-time cell analysis, replication kinetics). Immunostaining of infected cells was performed with MAb 1017, a monoclonal antibody directed against the HMPV F protein (a gift from MedImmune), followed by peroxidase-labeled goat anti-hamster immunoglobulin (Cederlane) and TruBlue peroxidase substrate (KPL/Mandel) as previously described [22].

### Construction of antigenome- and supporting protein plasmids

A pSP72 plasmid (Promega) was used to generate the antigenome plasmids as previously reported [23]. Briefly, an NdeI to HpaI fragment was removed from plasmid pSP72 (Promega) and replaced by a T7 terminator, the hepatitis delta virus (HDV) ribozyme and a T7 promoter to yield pSP72-T7_T_-δ-T7_P_. cDNA was generated from viral RNA using the Superscript II reverse transcriptase (Life technologies). PCR was carried out using PFU turbo polymerase (Life Technologies). The cDNA encoding the antigenome of C-85473 or CAN98–75 was assembled from 3 or 4 PCR fragments, cloned into temporary pJET plasmids (Thermo Scientific) and sequenced before being cloned into the pSP72 plasmid. The GFP gene was flanked by the N gene start region and the F gene end region of the respective strains and inserted between the N gene and the antigenomic leader sequence using the restriction sites MluI and StuI for CAN98–75 and MluI and NheI for C-85473. Subsequently, the F genes were interchanged between the 2 stains by site-directed mutagenesis using the Phusion DNA polymerase (New England Biolabs) in the case of CAN98–75_F and using the commercial Gibson Assembly Cloning Kit (New England Biolabs) in the case of C-85473_F (S1 Fig.).

The N, P, L, and M2.1 ORFs of CAN98–75 were amplified by PCR using primers spanning the start and stop codons and flanked by XhoI and NotI restriction sites and were subsequently cloned in the multiple cloning site of the pT_N_T vector (Promega) to create supporting protein plasmids.

All plasmids were sequenced using the ABI 3730 DNA analyzer and analyzed using BioEdit, version 7.2.0 prior to further use.

### Recombinant virus rescue

BSR-T7 cells were co-transfected with the plasmid containing the antigenome and the 4 supporting protein plasmids using Lipofectamin 2000 (Life Technologies) for 5 h, after which time the medium was replaced by Optimem + 1% NEAA. Transfected cells were incubated at 37°C and 5% CO_2_ until GFP expression was observed using fluorescent microscopy. At this point, LLC-MK2 cells were added to the transfected BSR-T7 cells and co-cultured for 2 to 3 days at 37°C and 5% CO_2_ with the addition of fresh trypsin (0.0002%) every other day. When infection was observed, cells were harvested, sonicated and centrifuged. The supernatant was then used to infect confluent LLC-MK2 monolayers and virus was cultured until CPE appeared. After 2 passages in LLC-MK2 cells, the recombinant viruses were concentrated using Amicon columns and an aliquot was used to verify the sequence of the F gene (GenBank accession numbers: KM408076.1 and AY145289.1 for C-85473 and CAN98–75, respectively).

### Syncytium quantification

Confluent monolayers of LLC-MK2 cells in black 24-well plates with flat and clear bottom (ibidi) were infected with each of the recombinant GFP-expressing HMPV viruses at an MOI of 0.01 in quadruplicate. Trypsine (0.0002%) was added every other day. Syncytium formation was evaluated on a daily basis using a fluorescent microscope. Three photographs were taken of each infected well at 20x magnification. In each field, 40 nuclei were counted and the number of nuclei per cell was calculated.

### Real time cell analysis

Real time cell analysis (RTCA) was performed using the xCELLigence System (ACEA). Fifty μl of cell culture medium was added to each well of a 96-well E-Plate (ACEA) to obtain background readings. LLC-MK2 cells were then added at 12 500 cells per well in 100 μl of culture medium. The E-Plates were subsequently incubated for 30 min at room temperature and placed on the RTCA MP station (ACEA) located in an incubator (at 37°C and 5% CO_2_). The Cell Index (CI) values were measured automatically every 30 min. When CI reached a plateau (24 h after seeding), cells were washed 2 times with 200 μl of PBS and 6 wells were infected with 150 μl of each of the recombinant HMPV viruses at an MOI of 0.01. Infected E-plates were placed back into the RTCA MP station and the CI values were measured automatically every 30 min for 7 days. Trypsin (0.0002%) was added every other day. Cell indexes were normalized to mock-infected wells and the time until CI was reduced by 50% was calculated for each virus.

### In vitro replication kinetics assay

Confluent monolayers of LLC-MK2 cells in 24-well plates were washed twice with PBS and infected with recombinant HMPV viruses at an MOI of 0.01. Trypsin (0.0002%) was added every other day. Three infected wells were harvested every 24 h for 7 days and supernatants were stored at -80°C. End-point titrations were performed on all samples to determine viral titers reported as TCID_50_/ml.

### Animal studies

Four week-old BALB/c mice (Charles River Laboratories) were housed in groups of three or four per micro-isolator cage. The mice were infected intranasally with 1x10^6^ TCID_50_ of clinical C-85473 and CAN98–75 HMPV strains or 6x10^5^ TCID_50_ of recombinant rC-85473, rCAN98–75, rC-85473_F and rCAN98–75_F HMPV strains. The animals were monitored on a daily basis for weight loss and the presence of clinical signs such as reduced activity and ruffled fur. Animals were sacrificed when they reached 20% weight loss. For experiments with recombinant HMPV strains, four mice per group were euthanized on days 3 through 6 pi using sodium pentobarbital and lungs were removed for the evaluation of viral titers by cell culture and for the evaluation of cytokine levels using a bead-based multiplex immunoassay. Finally, on day 5 pi, four more mice per group were euthanized and lungs were removed for histopathological analysis. The animal studies were approved by the Animal Protection Committee of the Centre Hospitalier Universitaire de Québec according to the guidelines of the Canadian Council on Animal Care.

### In vivo replication kinetics assay

Lungs were removed on days 3 through 6 pi, weighed and homogenized in 1 ml of PBS then centrifuged at 2000 rpm for 10 min. The supernatant was used to determine viral titers reported as TCID_50_/g of lung.

### Cytokine analysis

An aliquot of 150 μl of lung homogenates was added to 150 μl of 50 mM KPO_4_, pH 6.0 buffer containing 0.2% CHAPS (Sigma) and 0.2% protease inhibitor cocktail (Sigma) and then stored at -20°C. On the day of the experiment, samples were centrifuged at 13,000 × g for 10 min at 4°C and then 50 μl of the supernatant were used for cytokine quantification using a commercial multiplex mouse cytokine bead assay (Bio-Rad) according to the manufacturers’ instructions. Experiments were performed in flat bottom 96-well plate and results were analyzed with the Luminex system (QIAGEN).

### Histopathological analysis

Lungs were removed on day 5 pi, and fixed with 4% buffered formalin. Fixed lungs were subsequently embedded in paraffin, sectioned in slices of 5 μm, and stained with hematoxylin-eosin. Scoring of histologic parameters was performed by a medical biologist (SL) and an anatomic pathologist (CC), both with experience in pulmonary pathology, independently and blind to experimental data, on digitalized slides scanned at a resolution of 400X magnification (Nanozoomer scanner and viewer, Hamamatsu, Japan). A semi-quantitative scale was used to score bronchial/endobronchial, peribronchial, perivascular, interstitial, pleural and intra alveolar inflammation, capillary vascular congestion and pulmonary edema [21].

### Statistical analysis

Repeated-Measure ANOVA’s with tukey post-hoc tests were used to analyze all the data except those involving RTCA for which two-tailed Student t-tests were used. All statistical analyses were performed using Prism 6.

## Results

### *In vitro* and *in vivo* properties of two clinical HMPV strains

**Fig 2:**
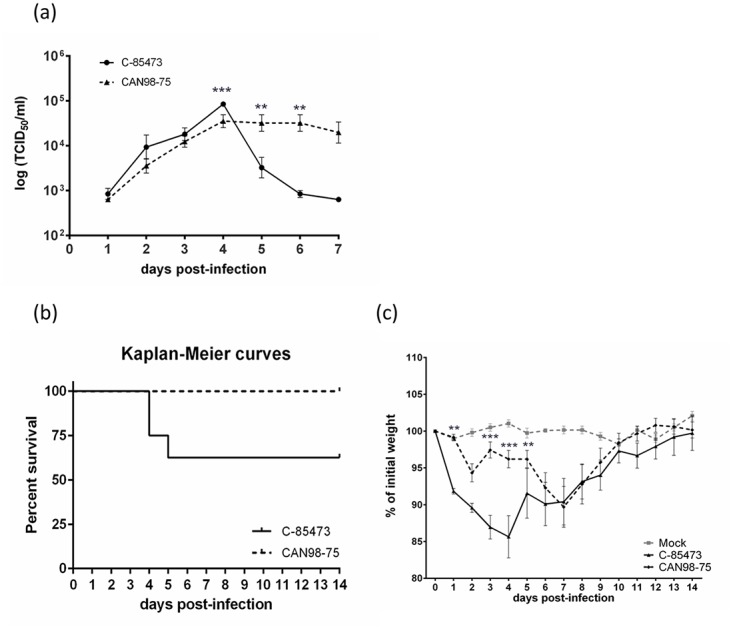
Replicative capacity of the clinical HMPV isolates C-85473 and CAN98–75 *in vitro* and virulence in BALB/c mice. (a) Replicative capacity of C-85473 and CAN98–75 strans in LLC-MK2 cells at an MOI of 0.1. (b) Kaplan-Meier survival curves of mice infected with 1x10^6^ TCID_50_ of C-85473 or CAN98–75. (c) Weight loss curves of BALB/c mice infected with 1x10^6^ TCID_50_ of C-85473 or CAN98–75 or mock infected. **, p < 0.01; ***, p < 0.001 comparing C-85473 to CAN98–75 using Repeated Measures Two-way ANOVA.

LLC-MK2 monolayers were infected with either C-85473 or CAN98–75 viruses at an MOI of 0.1 to evaluate *in vitro* replication kinetics. The syncytium-inducing C-85473 strain replicated to a significantly higher titer than the focal cell rounding strain CAN98–75 on day 4 pi (8.6 ± 0.8 vs 4.2 ± 1.3 x10^4^ TCID_50_/ml; p<0.001) (Fig. 2a). After day 4, however, viral titers of strain C-85473 decreased more rapidly than those of the non-syncytium inducing strain.

Intranasal infection of BALB/c mice with 10^6^ TCID_50_ of C-85473 led to 37.5% mortality by day 5 post-infection (pi) whereas infection with the same inoculum of CAN98–75 did not induce any mortality (Fig. 2b). Mice infected with C-85473 reached their maximum weight loss on day 4 pi (14.4 ± 2.9%), whereas CAN98–75-infected mice reached their maximum weight loss much later, on day 7 pi (10.3 ± 2.8%) (Fig. 2c). Furthermore, pulmonary viral titers were determined by cell culture on day 5 pi. The C-85473 strain replicated to higher pulmonary viral titers compared to CAN98–75 (18.3 ± 4.6 vs 3.8 ± 1.3x10^3^ TCID_50_/g lung; p<0.01).

### Generation of recombinant HMPV strains

Based on these observations, we postulated that the F protein, leading to syncytium formation, was also responsible for the increase in HMPV virulence. Thus, we generated recombinant viruses for each strain and swapped the F proteins of both viruses in order to investigate the contribution of individual F proteins in each viral background. Full-length antigenome-plasmids were created for rC-85473, rCAN98–75, rC-85473_F and rCAN98–75 (S1 Fig.). Following co-transfection of the antigenome-plasmids and supporting plasmids into BSR T7/5 cells, GFP expression was observed on average 48 h post-transfection. LLC-MK2 cells were then added to amplify viral production and recombinant viruses were rescued within 5 days of transfection.

### *In vitro* phenotype of four recombinant HMPV strains

**Fig 3:**
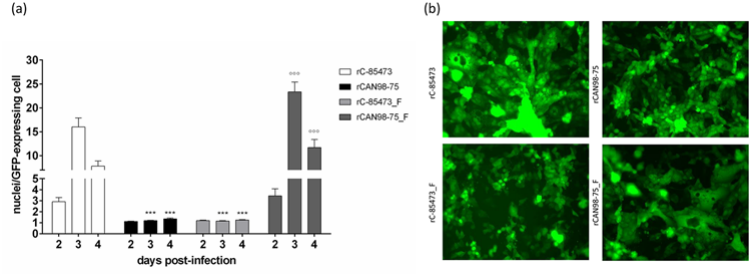
Syncytium formation induced by recombinant HMPV strains. (a) LLC-MK2 monolayers in 24 well-plates were infected with rHMPV at an MOI of 0.01 in quadruplicate. On days 2 through 4 pi, pictures were taken using fluorescent microscopy in 3 random fields (20x magnification) per well and the number of nuclei per GFP-expressing cell was calculated. ***, p < 0.001 comparing all other strains to rC-85473 and °°°, p < 0.001 comparing all other strains to rCAN98–75 using Repeated Measures Two-way ANOVA. (b) An example of the observed difference in syncytium formation between the 4 recombinant strains on day 3 pi.

The *in vitro* phenotype of each HMPV recombinant virus was investigated using fluorescence microscopy. The wild-type (WT) C-85473 strain is known to induce large syncytia and the recombinant strain rC-85473 conserved the same phenotype with 15.9 ± 1.9 nuclei per GFP-expressing cell on day 3 p.i (Fig. 3). On the other hand, the recombinant rCAN98–75 induced mostly focal cell rounding, similar to its WT counterpart with 1.2 ± 0.04 nuclei per GFP-expressing cell on day 3 p.i. When the F gene of rC-85473 was replaced with that of rCAN98–75 (rC-85473_F), the phenotype was reverted and less syncytia were observed with 1.2 ± 0.05 nuclei per GFP-expressing cell on day 3 p.i. Finally, rCAN98–75_F induced more syncytia with 23.3 ± 2.1 nuclei per GFP-expressing cell on day 3 p.i. These data confirmed that syncytium formation was primarily dependent on the F gene of the strain C-85473.

### Effects of four recombinant HMPV strains on cell state

**Fig 4:**
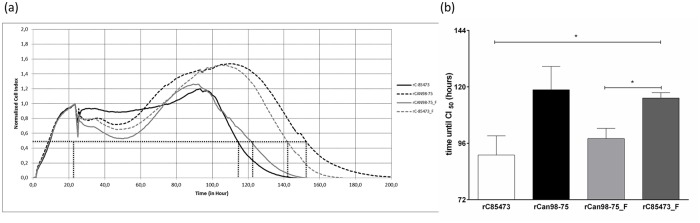
Real-time cell analysis of recombinant HMPV strains. LLC-MK2 monolayers in 96 well-plates were infected with rHMPV at an MOI of 0.01 (a) Output of one real-time cell analysis (RTCA) experiment; data was normalized using mock-infected wells and normalized cell index is plotted. (b) Mean time until cell index is reduced by 50% from 4 independent experiments is plotted. *, p < 0.05 using unpaired, two-tailed Student t-test.

We also investigated the effect of HMPV infection on the state of LLC-MK2 monolayers using RTCA. This method measures the change in electrical impedance across a cell monolayer in real-time. A parameter called cell index (CI) is used to quantify cell status based on the detected cell-electrode impedance; an elevated CI means that cells have fully adhered to the well and have proliferated, whereas a low CI indicates changes in morphology and viability of the cell monolayer. The changes in CI during an infection experiment using RTCA is shown in Fig. 4a and mean time until the normalized CI was reduced by 50% is reported in Fig. 4b. On average, it took rCAN98–75 28 h longer to reduce the CI by 50% than rC-85473 ((118 ± 9 h compared to 91 ± 8 h). Exchanging the F protein resulted in reverse phenotypes with 50% reduction of CI obtained by 98 ± 4 h and 115 ± 2 h for rCAN98–75_F and rC-85473_F, respectively. These data show that the HMPV strains carrying the syncytium-inducing F protein from C-85473 alter the cell state of infected cells faster than viruses carrying the F protein from CAN98–75.

### *In vitro* replication kinetics assay

**Fig 5:**
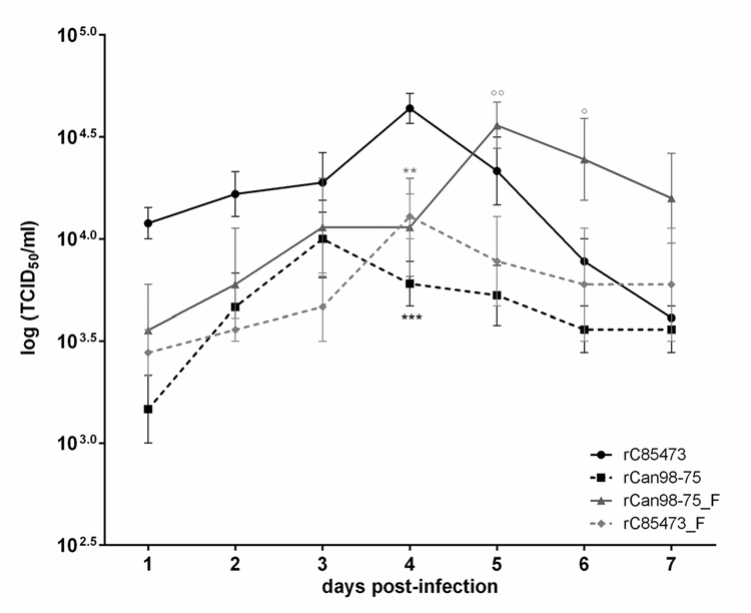
*In vitro* replicative capacity of recombinant HMPV strains. LLC-MK2 monolayers in 24 well-plates were infected with 4 rHMPV strains at an MOI of 0.01 in triplicate, one well per condition was harvested every 24 h for 7 days, frozen, sonicated and titrated on LLC-MK2 cells. ***, p < 0.001; ** p < 0.01 comparing all other strains to rC-85473 and °°, p < 0.01; °, p < 0.05 comparing all other strains to rCAN98–75 using Repeated Measures Two-way ANOVA.

We next sought to investigate the replicative capacity of each recombinant virus *in vitro*, using an MOI of 0.01 (Fig. 5). Over a 7-day period, rC-85473 and rCAN98–75_F (maximum titers of 4.5 ± 0.7 and 3.8 ± 0.8 x10^4^ TCID_50_/ml, respectively) replicated to significantly higher titers (p<0.05) than rCAN98–75 and rC-85473_F (maximum titers of 1.2 ± 0.5 and 1.3 ± 0.4 x10^4^ TCID_50_/ml, respectively). Of note, chimeric viruses (rCAN98–75_F and rC-85473_F) reached their peak titers 24 h later than recombinant WT viruses (rC-85473 and rCAN98–75). This experiment confirms that syncytium-inducing strains replicate to higher viral titers than non-syncytium inducing strains.

### Replication kinetics of HMPV strains in lungs of BALB/c mice

**Fig 6:**
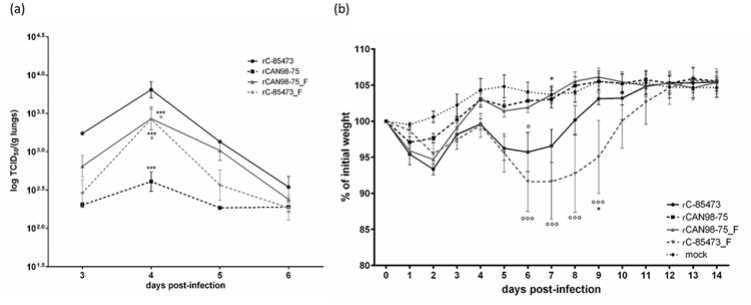
Lung viral titers and weight loss of BALB/c mice infected with recombinant HMPV strains. BALB/c mice were infected with 6x10^5^ TCID_50_ of rHMPV (as determined by backtitration). (a) On days 3 through 6, four mice per group were euthanized to determine pulmonary viral titers. (b) Six mice per group were monitored for weight loss on a daily basis for 14 days. ***, p < 0.001; * p < 0.05 comparing all other strains to rC-85473.°°°, p < 0.001; °, p < 0.05 comparing all other strains to rCAN98–75 using Repeated Measures Two-way ANOVA.

To investigate the replicative capacity of all 4 recombinant viruses *in vivo*, BALB/c mice were infected with 6x10^5^ TCID_50_ of recombinant HMPV strains. Backtitrations of the inoculum confirmed that the same amount of recombinant HMPV was given to all groups (6.3, 6.0, 6.8 and 6.4 x 10^5^ TCID_50_/mouse for rC-85473, rCAN98–75, rCAN98–75_F and rC-85473_F, respectively). Such inoculum did not induce mortality in any of the groups. Lungs of infected mice were harvested on day 3 through 6 post-infection to determine viral titers. All 4 recombinant viruses reached their peak of replication on day 4 pi (Fig. 6a). rC-85473 replicated to the highest titer (7.2 ± 2.1 x10^3^ TCID_50_/g lung) whereas rCAN98–75 had the lowest peak (4.6 ± 1.3 x10^2^ TCID_50_/g lung). Conversely, the chimeric strains rCAN98–75_F and rC-85473_F replicated to similar peak titers (3.2 ± 1.01 and 3.0 ± 0.9 x10^3^ TCID_50_/g lung, respectively).

### Weight loss of HMPV-infected mice

Mice infected with 6x10^5^ TCID_50_ of recombinant HMPV strains or mock infected were monitored for 14 days for clinical signs and weight loss. All infected mice lost between 3 and 7 percent of their initial weight between days 1 and 3, but only viruses with the C-85473 background (rC-85473 and rC85473_F) continued to lose weight on days 5–7, with statistically significant differences observed between rC-85473-viruses and rCAN98–75 viruses on days 6–9 (Fig. 6b). No significant difference in weight loss was observed between the 2 viruses with CAN98–75 background (rCAN98–75 and rCAN98–75_F). rC-85473-infected mice appeared to recuperate a little bit faster than mice infected with rC-85473_F with a statistically significant difference observed on day 9 pi only. Thus, the severity of the HMPV symptoms correlated better with the viral background than the F protein.

### Pulmonary cytokine levels of HMPV-infected mice

**Fig 7:**
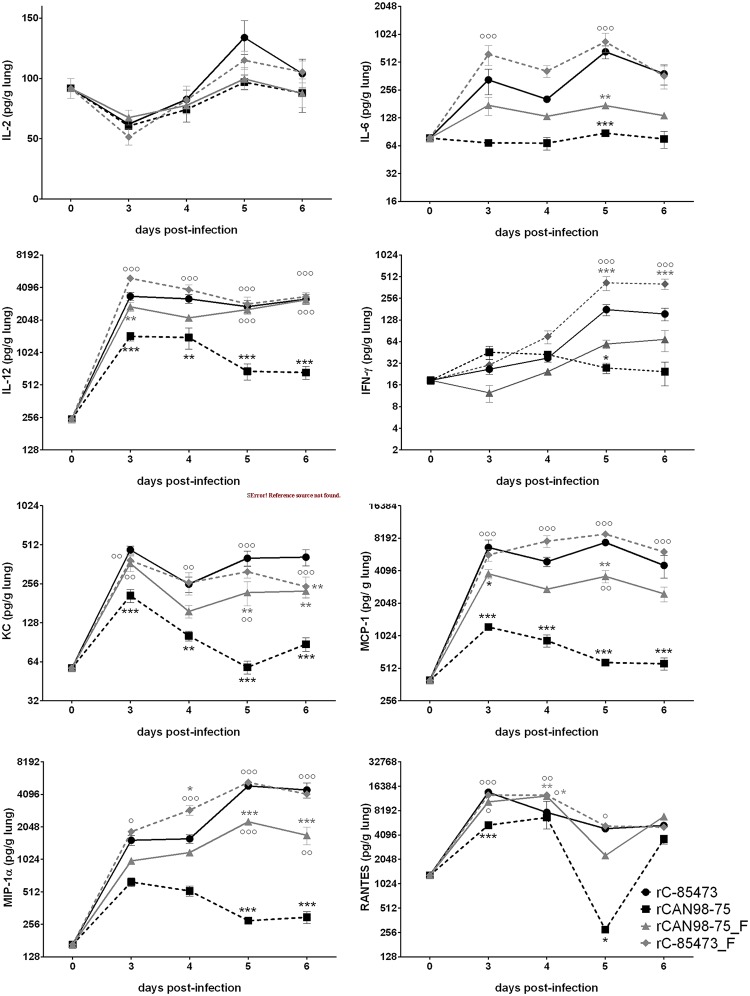
Pulmonary cytokine levels of BALB/c mice infected with recombinant HMPV strains. BALB/c mice were infected with 6x10^5^ TCID_50_ of rHMPV (as determined by back-titration). On days 3 through 6, four mice per group were euthanized to determine pro-inflammatory cytokine/chemokine levels in the lungs of infected mice. Mock-infected mice are representad as day 0. ***, p < 0.001; **, p < 0.01; * p < 0.05 comparing all other strains to rC-85473.°°°, p < 0.001; °°, p < 0.01; °, p < 0.05 comparing all other strains to rCAN98–75 using Repeated Measures Two-way ANOVA.

On days 3 through 6 pi of the previously described experiment, an aliquot of lung homogenates was used to determine pulmonary cytokine/chemokine levels using a multiplexed bead assay (Fig. 7).

IL-2 peaked on day 5 pi for all 4 recombinant viruses and IL-2 levels were similar between groups at each time point. IL-6 also peaked on day 5 pi, but IL-6 levels were significantly higher in viruses with a C-85473 background compared to viruses with a CAN98–75 background on days 5 pi. IL-12 levels remained relatively stable between days 3 and 6 pi, except for rCAN98–75 for which IL-12 levels were reduced by half by day 5 pi. Significantly higher levels of IL-12 were observed with the viruses harboring the C-85473 background compared to the rCAN98–75 virus on all analyzed time-points. However, introducing the F protein of C-85473 into rCAN98–75 significantly increased IL-12 levels on days 5 and 6 pi. IFN-γ levels reached a plateau on days 5 and 6 pi for both viruses with a C-85473 background as well as for rCAN98–75_F, whereas IFN-γ levels declined from day 3 onward for rCAN98–75. KC levels were highest on day 3 pi, with a second peak on day 5 pi for all viruses except rCAN98–75, for which KC levels increased a day later. KC levels were significantly higher for recombinant viruses with a C-85473 background compared to rCAN98–75. Furthermore, the introduction of the F protein from C-85473 into the CAN98–75 background significantly increased KC levels on days 3, 5 and 6 pi compared to the wild-type rCAN98–75.

MCP-1 levels were consistently significantly higher for viruses with a C-85473 background compared to rCAN98–75. Moreover, on day 5 pi, a significantly higher MCP-1 level was observed in mice infected with rCAN98–75_F compared to rCAN98–75. MIP-1α levels peaked on day 5 pi for all strains except rCAN98–75, which peaked on or before day 3 pi. Furthermore, significantly higher MIP-1α levels were observed on days 5 and 6 pi for rC-85473 and rC-85473_F as well as for rCAN98–75_F, compared to rCAN98–75. In general RANTES levels were lower for strain rCAN98–75 than for the other 3 viruses with statistically significant differences observed at early time-points.

### Lung histopathology studies of HMPV-infected mice

**Fig 8:**
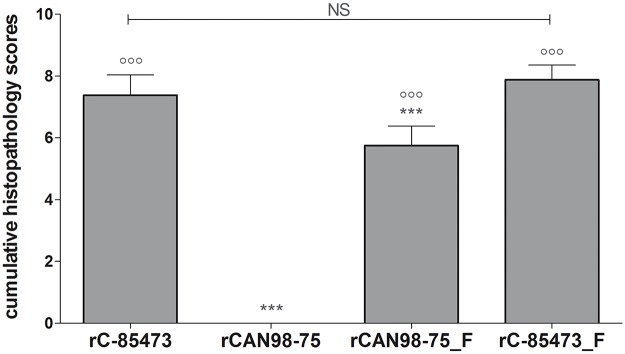
Total pulmonary inflammation scores of BALB/c mice infected with recombinant HMPV strains. BALB/c mice were infected with 6x10^5^ TCID_50_ of rHMPV (as determined by back-titration). On days 5, four mice per group were euthanized to determine histopathology scores of the lungs of infected mice. ***, p < 0.001; comparing all other strains to rC-85473.°°°, p < 0.001 comparing all other strains to rCAN98–75 using Repeated Measures Two-way ANOVA.

On day 5 of the previously described experiment, lungs were harvested from 4 more mice per group to assess pulmonary inflammation. None of the groups showed signs of vascular congestion, pulmonary edema or bronchial inflammation. Moreover, none of the mice infected with rCAN98–75 showed signs of pulmonary inflammation for any of the analyzed parameters. Conversely, mice infected with rC-85473 or rC-85473_F showed mild, moderate or moderate to marked scores, with no significant differences in scores for any of the parameters between these two groups (total inflammation scores of 7.4 ± 0.7 and 7.9 ± 0.5 for rC-85473 and rC-85473_F, respectively) (Fig. 8). Importantly, the introduction of the syncytium-inducing F protein into the rCAN98–75 background significantly increased histopathology scores for peribronchial, perivascular, interstitial and intra-alveolar inflammation (data not shown) as well as total inflammation (total inflammation score of 5.8 ± 0.6). In summary, although the C-85473 background induced significantly more pulmonary inflammation, the introduction of the syncytium-inducing F protein into the rCAN98–75 background significantly increased lung inflammation.

## Discussion

In the current study, we examined the effects of the F protein from two different HMPV strains, producing large syncytia in cell culture or not, on *in vitro* and *in vivo* replication kinetics and virulence. We generated recombinant HMPV viruses representing either the syncytium-inducing phenotype (strain rC-85473) or the focal cell rounding phenotype (strain rCAN98–75) and we subsequently exchanged the F genes of both strains. We demonstrated that syncytium phenotype mainly depends on the F protein and that viruses carrying an F protein that induces syncytium-formation replicate to higher titers *in vitro*. However, although the F protein appears to contribute to HMPV virulence, other genetic markers within the HMPV genome seem to impact on disease severity in mice.

HMPV is an important respiratory pathogen that can cause upper and lower RTIs. Virological risk factors for severe HMPV disease, such as HMPV subtype or lineage, have been the object of several investigations, with conflicting results [6,8–10,24]. We first sought to investigate whether the *in vitro* phenotype, namely syncytium formation, might be an indication of efficient HMPV replicative capacity. Previously, the syncytium-inducing strain NL/1/99 (subtype B1) was found to replicate to higher titers in Vero-118 cells than NL/1/00 (subtype A1), a strain that does not produce syncytium at neutral pH [23,25]. Similarly, we found that the syncytium-inducing strain C-85473 replicated to higher titers in LLC-MK2 cells than the focal cell rounding strain CAN98–75. However, to our knowledge, the impact of *in vitro* phenotype on *in vivo* replication i.e., on lung titers, had not yet been examined. For this purpose, we infected BALB/c mice with either C-85473 or CAN98–75 clinical isolates and found that the former replicated to higher titers in the lungs on day 4 pi than CAN98–75 in 3 independent experiments (data from one representative experiment are shown in Fig. 2). Furthermore, mortality was only observed in mice infected with strain C-85473.

Similarly to other paramyxoviruses, HMPV enters the host cell by fusion of viral and cellular membranes, a step mediated by surface glycoproteins. On its surface, HMPV carries 3 glycoproteins (F, G and SH) of which the F protein is the most conserved among HMPV strains [12]. As such, the HMPV F protein also shares structural features with other paramyxovirus F proteins; it is a class I viral fusion protein synthesized as inactive precursors (F_0_) that must be cleaved into 2 disulfide-linked F_2_-F_1_ subunits to be fusion-competent [15]. Unlike members of the *Paramyxovirinae* subfamily, but similarly to other members of the *Pneumovirinae* subfamily including HRSV, the HMPV F protein mediates membrane fusion in the absence of a separate viral attachment protein [16,26]. Furthermore, by transfecting cells with recombinant HMPV F proteins, it was demonstrated that the F protein alone is able to induce syncytium formation in cell culture [16,17].

To investigate the role of the F protein on viral replication both *in vitro* and *in vivo*, we used reverse genetics to generate GFP-expressing recombinant viruses with either the C-85473 or the CAN98–75 genome and we further exchanged the F genes of both strains. Inserting the F gene of C-85473 into the CAN98–75 background restored the syncytium phenotype of strain C-85473, confirming that the F protein is responsible for syncytium formation independently of the genomic background.

Moreover, using RTCA, we demonstrated that the syncytium-inducing rHMPV strains induced changes in cell state 24 h before the focal cell rounding viruses. RTCA is a new electronic cell sensor array, in which the impedance, displayed as CI values, is continuously measured to evaluate cellular integrity [27,28]. This parameter includes cell proliferation, adhesion, viability, morphology and motility. This novel technique has been used to evaluate CPE and their inhibition by neutralizing antibodies directed against flaviviruses [28] and influenza A viruses [27], but also to evaluate antiviral activity [29,30] and cytotoxicity [31]. Here, we demonstrate the usefulness of RTCA in investigating the dynamics of HMPV infection for the first time.

A significant increase in *in vitro* replicative capacity of syncytium-inducing HMPV strains was also confirmed using replication kinetics assays. Laboratory strains were passaged extensively (10 times), which increased their viral titers significantly. On the other hand, we only passaged the recombinant viruses twice to avoid introducing mutations and defective interfering RNAs that could alter the immune response *in vivo* [32], but this led to lower viral stock titers (by about 1 log). Although the change in genomic background did not alter peak viral titers of viruses expressing the same F protein, both chimeric viruses reached their peak of replication a day later than WT rHMPV strains; this could indicate some influence of the genomic background on F protein incorporation and/or expression.

Notably, we investigated whether *in vitro* phenotypes also correlated with *in vivo* replication titers. We observed that peak lung titers occurred on day 4 pi for all recombinant HMPV viruses. Again, rC-85473 was associated with the highest viral titers and introducing the F protein of CAN98–75 into this strain significantly reduced peak viral titers (Fig. 6a). Conversely, the rCAN98–75 strain generated the lowest peak viral titers and introducing the F protein of C-85473 significantly increased maximum pulmonary viral titers. However, the overlap in the replication curves of the two chimeric viruses suggests that *in vivo* properties were not exclusively dependent on the F protein. This was even more evident by looking at weight loss curves where strains were primarily segregated by their genomic background and not by their F protein. Similarly, pro-inflammatory cytokine/chemokine levels and pulmonary inflammation scores were increased in the rC-85473 strains independently of the F protein, although rCAN98–75_F induced higher cytokine levels (particularly MIP-1α, MCP-1, IL-12 and KC) and significantly more pulmonary inflammation than the prototypic rCAN98–75 strain, suggesting that the F protein does have an effect on HMPV pathogenesis as well.

We investigated 8 cytokines/chemokines (IL-2, IL-6, IL-12, IFN-γ, KC, MIP-1α, MCP-1 and RANTES) that had previously been described to be up-regulated in the lungs of HMPV-infected mice [21,33,34]. Although different inoculums and cytokine detection methods were used in previously-published studies, we also observed up-regulation of all cytokines/chemokines in mice infected with C-85473 strains, but only MIP-1α and MCP-1, two chemokines involved in the recruitment and activation of leukocytes, as well as IL-12, a cytokine involved in Th1 differentiation, were significantly up-regulated for rCAN98–75 compared to mock-infected mice (represented as day 0 on the graph).

Unlike HRSV, HMPV does not encode non-structural proteins known to inhibit antiviral immune responses. Therefore, other viral proteins must be involved in immune evasion mechanisms. Among HMPV-expressed proteins, the SH and G glycoproteins, the P phosphoprotein and the M2–2 protein have been found to have immune evasive properties. The presence of the SH protein reduced the expression of TNF-α, IL-6, KC and MCP-1 in mice infected with WT HMPV compared to mice infected with HMPV lacking the SH protein, in an NF-κB-dependent manner [35]. The G protein was found to reduce cytokine/chemokine levels in cell cultures by inhibiting RIG-1 signaling [36,37]. Furthermore, the G protein inhibited TLR4-signaling in dendritic cells [38]. Goutagny et al. observed that a HMPV-B1 strain impaired type I IFN production, specifically by prevented RIG-I-mediated sensing of HMPV viral RNA, in a P protein-dependent manner [39]. Finally, the M2–2 protein inhibited MAVS-induced IFN-β gene transcription *in vitro* [40]. One or several of these viral proteins may be implicated in the observed differences in pathogenesis between strains with the C-85473 and CAN98–75 genomic background and this will be further investigated.

We did not investigate pH-dependency of our different HMPV strains. In contrast to previous reports [17,18], our syncytium-inducing virus is a subtype A strain and the focal cell rounding virus is a subtype B strain. This suggests that, although low-pH-triggered syncytium-formation may be subtype-dependent, syncytium formation at neutral pH does not appear to be subtype-dependent. Furthermore, the physiological relevance of low pH-dependent syncytium-formation remains to be elucidated. Interestingly, our large syncytium-inducing strain carries EKRN at the proposed tetrad positions of the F protein, which are the same as NL/17/00, a poorly fusogenic strain [18]. Moreover, our focal cell-rounding strain carries EDRP, the same tetrad as NL/1/94, a pH-independent syncytium-inducing strain [18]. This suggests that residues at other positions are likely to influence syncytium formation at neutral pH. Given the importance of the heptad repeats located in F_1_ subdomain of the F protein for viral fusion, it would be interesting to examine the effect of the five amino acid changes in HRA and the two amino acid changes in HRB that differentiate our two prototype strains, on syncytium formation at neutral pH.

In conclusion, we have demonstrated the importance of the HMPV F protein for syncytium formation and *in vitro* replication and further showed that the F protein contributes to some extent, but not exclusively, to the virulence potential of different strains in mice. Therefore, it is unlikely that the *in vitro* phenotype of an HMPV strain alone is sufficient to predict the severity of HMPV disease. Other syncytium-inducing and non-syncytium inducing HMPV strains of different subtypes should be studied to confirm our results. Furthermore the effects of other viral genes on HMPV pathogenesis should be investigated using recombinant viruses in different animal models.

## Supporting Information

S1 Fig Construction of HMPV antigenome plasmids. Schematic representation of the cloning steps for rCAN98–75 (a) and for rC-85473 (b). Three or four cDNA fragments (A-C for rC-85473 and A-D for rCAN98–75) were cloned into the pSP72-T7_T_-δ-T7_P_ vector. The obtained antigenome plasmids were then used to swap the F genes. (c) Schematic representation of the cloning steps used to obtain rCAN98–75_F; a fragment covering M to SH was amplified from the rCAN98–75 antigenomic plasmid and cloned into the temporary pJET vector. Simultaneously, the F gene was amplified from C-85473 cDNA and also cloned into pJET. Both plasmids were digested and the F gene was ligated into the temporary pJET plasmid. From this vector, a fragment covering the region M to M2 was amplified and used as primers for site-directed mutagenesis of the rCAN98–75 antigenomic plasmid. (d) A schematic representation of the cloning steps used to obtain rC-85473_F; a fragment containing the region P to M2 of rC-85473 was cloned into a temporary pJET vector. A fragment of the F gene of rCAN98–75, flanked by the restriction sites EcoRV and PacI, was amplified by PCR and subsequently cloned into the temporary pJET vector containing the rC-85473 fragment. From this vector, fragment 1 was amplified by PCR. Fragment 2 was amplified directly from the plasmid containing the rCAN98–75 antigenomic vector and fragments 3 and 4 were amplified from the rC-85473 antigenomic vector. Finally the pSP72 vector was digested to obtain fragment 5. All 5 fragments were then ligated using the Gibson Assembly cloning kit.
(TIF)
