# Supplementary figures and images for: Effect of In Vitro Syncytium Formation on the Severity of Human Metapneumovirus Disease in a Murine Model
Source: PLoS One. 2015 Mar 24;10(3):e0120283. doi: 10.1371/journal.pone.0120283 (PMC4372586; doi:10.1371/journal.pone.0120283)

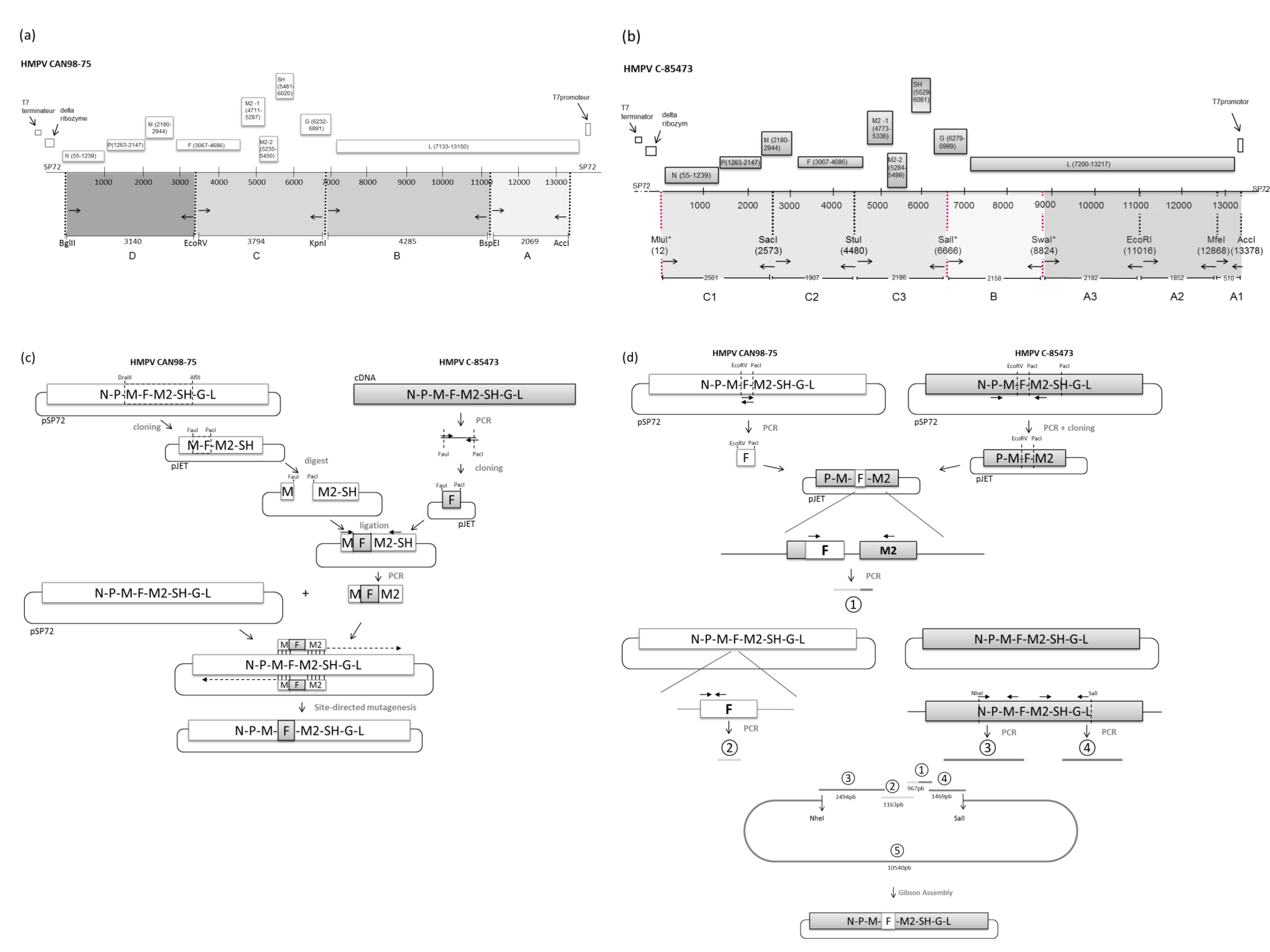

Supplement: S1 Fig — Schematic representation of the cloning steps for rCAN98–75 (a) and for rC-85473 (b). Three or four cDNA fragments (A-C for rC-85473 and A-D for rCAN98–75) were cloned into the pSP72-T7T-δ-T7P vector. The obtained antigenome plasmids were then used to swap the F genes. (c) Schematic representation of the cloning steps used to obtain rCAN98–75_F; a fragment covering M to SH was amplified from the rCAN98–75 antigenomic plasmid and cloned into the temporary pJET vector. Simultaneously, the F gene was amplified from C-85473 cDNA and also cloned into pJET. Both plasmids were digested and the F gene was ligated into the temporary pJET plasmid. From this vector, a fragment covering the region M to M2 was amplified and used as primers for site-directed mutagenesis of the rCAN98–75 antigenomic plasmid. (d) A schematic representation of the cloning steps used to obtain rC-85473_F; a fragment containing the region P to M2 of rC-85473 was cloned into a temporary pJET vector. A fragment of the F gene of rCAN98–75, flanked by the restriction sites EcoRV and PacI, was amplified by PCR and subsequently cloned into the temporary pJET vector containing the rC-85473 fragment. From this vector, fragment 1 was amplified by PCR. Fragment 2 was amplified directly from the plasmid containing the rCAN98–75 antigenomic vector and fragments 3 and 4 were amplified from the rC-85473 antigenomic vector. Finally the pSP72 vector was digested to obtain fragment 5. All 5 fragments were then ligated using the Gibson Assembly cloning kit. (TIF) [file pone.0120283.s001.tif]
